# Size effect on thermal conductivity and stability of TiO_2_/MWCNT-based hybrid nanofluids synthesized *via* probe ultrasonication

**DOI:** 10.1039/d5ra06184a

**Published:** 2025-11-14

**Authors:** S. Heshmatian, M. Aligholami, S. Shafiei, I. G. Madiba, S. Azizi, Ahmed A. Hussein, M. Maaza

**Affiliations:** a UNESCO-UNISA Africa Chair in Nanosciences-Nanotechnology, College of Graduate Studies, University of South Africa Muckleneuk ridge, PO Box 392 Pretoria South Africa azizis@unisa.ac.za maazam@unisa.ac.za; b Nanosciences African Network, Materials Research Dept. iThemba LABS/National, Research Foundation of South Africa 1 Old Faure road, PO Box 722 Somerset West South Africa; c Department of Engineering Sciences and Physics, Buein Zahra Technical University Buein Zahra 3451866391 Iran; d Chemistry Department, Office 2-33, Cape Peninsula University of Technology, Bellville Campus Private Bag X17 Bellville 7535 Cape Town South Africa

## Abstract

This study reports the enhancement of thermal conductivity in hybrid TiO_2_ grafted onto multi-wall carbon nanotubes (MWCNTs) dispersed in an ethylene glycol nanofluid synthesized by a scalable probe-ultrasonication process. The hybrid nanofluids were formulated at ultra-low loadings; MWCNT = 0.001 wt% (fixed) and TiO_2_ = 0.001–0.01 wt% (15 nm and 30 nm). The 15 nm TiO_2_ sample at 0.01 wt% achieved 16.7% thermal conductivity enhancement at 70 °C while maintaining >4 weeks stability. To the best of our knowledge, this is the first report achieving double-digit conductivity improvement at ≤0.01 wt% solids using a surfactant-free, scalable probe-ultrasonication route. Homogeneous and stable TiO_2_/MWCNT nanofluids were produced using a surfactant-free approach, and their performance was validated through Raman spectroscopy, Zetasizer, TEM, and UV-Vis analyses. Formulations with ultra-low loadings, MWCNT = 0.001 wt% (fixed) and TiO_2_ = 0.001–0.01 wt% (15 or 30 nm), were investigated. The sample containing 15 nm TiO_2_ at 0.01 wt% exhibited a reproducible 16.7% thermal-conductivity enhancement at 70 °C and maintained colloidal stability for over four weeks. Such a high enhancement at extremely low solid content in an ethylene glycol matrix, achieved through a surfactant-free and scalable ultrasonication route, has not been previously reported.

## Abstract 2

A scalable, surfactant-free probe ultrasonication method produces stable TiO_2_/MWCNT hybrid nanofluids with 16.7% thermal conductivity enhancement at ultra-low concentrations.

## Graphical abstract

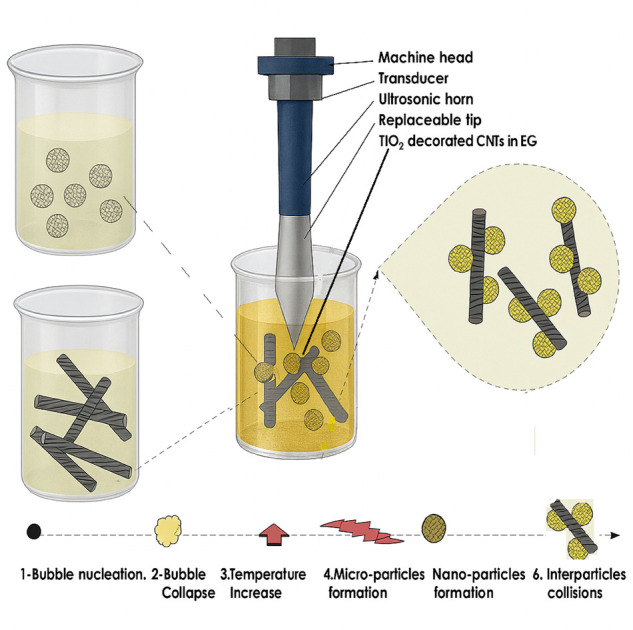

## 1. Introduction

**Fig. 1:**
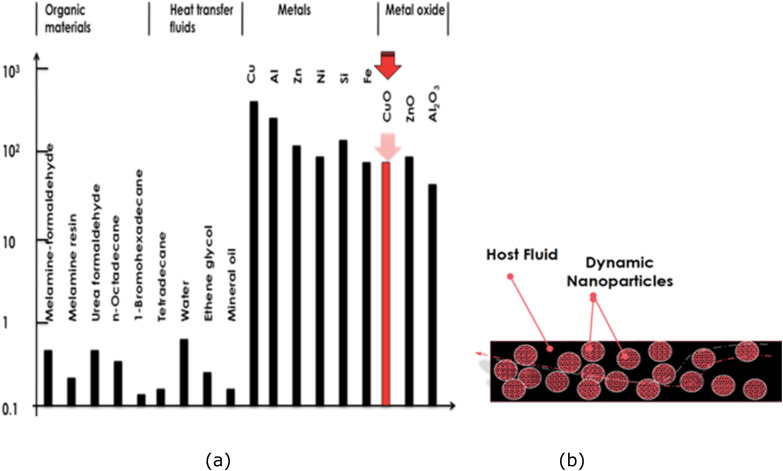
(a) Comparative scale of thermal conductivities of organic materials, standard heat-transfer fluids, and metallic/oxide solids. (b) Schematic representation of a nanofluid comprising suspended nanoparticles in a host fluid.

The efficiency of conventional heat-transfer fluids in industrial thermal-management systems is limited by their inherently low thermal conductivity (Fig. 1a). Typically, standard organic and heat-transfer fluids exhibit thermal conductivities below 1 W m^−1^ K^−1^, whereas metals and their oxides display values one to two orders of magnitude higher. As illustrated in Fig. 1b, nanofluids consisting of nanoscale particles dispersed in a host fluid represent a new generation of engineered coolants. Introducing nanoparticles into a base fluid is an effective strategy to overcome the limitations of traditional fluids and substantially improve their thermophysical properties.^1–3^

Over the past two decades, nanofluids have received considerable attention for their enhanced thermal conductivity and potential applications in solar energy harvesting, engine heat management, electronic and automotive cooling, data-center thermal control, and heat-exchanger design.^4–10^ Various preparation techniques have been developed, with one-step methods such as pulsed laser ablation in liquid (PLAL)^11–14^ and γ-radiolysis^15^ offering high-quality dispersions, though their scalability and cost remain challenging for large-scale implementation.

The objectives of this study are to:

(i) Validate the effectiveness of probe ultrasonication as a scalable approach for synthesizing hybrid TiO_2_-grafted MWCNT/ethylene glycol (EG) nanofluids;

(ii) Achieve thermal conductivity enhancements of ∼16% with stability exceeding four weeks; and.

(iii) Demonstrate the ultrasonication method as a sustainable, mass-production technique for hybrid nanofluids.

Although one-step techniques such as pulsed laser ablation and radiolysis yield stable dispersions and notable improvements in thermal conductivity, they remain constrained by high cost and poor scalability-key limitations for industrial application. In contrast, probe ultrasonication provides a simple, energy-efficient, and scalable route to disperse TiO_2_ decorated MWCNTs in ethylene glycol, effectively minimizing agglomeration and eliminating the need for surfactants. Its effectiveness arises from cavitation phenomena (bubble nucleation, collapse, and microjet formation), which generate intense localized energy and promote homogeneous nanoparticle dispersion.

Parameters such as Brownian motion, particle–fluid interactions, and agglomeration dynamics are crucial for understanding heat-transfer mechanisms in nanofluids.^16–20^ Properties including particle size, morphology, surface coordination, zeta potential, and base-fluid composition govern both heat conduction and long-term stability. Titanium dioxide (TiO_2_) nanoparticles, owing to their high thermal conductivity, chemical inertness, and low cost, have emerged as ideal candidates for nanofluid design.^21–33^ Numerous studies have investigated the effects of particle size, concentration, and base fluid on TiO_2_ nanofluids, reporting various degrees of conductivity enhancement (Table 1).

**Table 1:** Summary of relevant research on thermal conductivity of TiO_2_ nanofluids

References	Average particle size	Hybrid material (if any)	Base fluid	Thermal conductivity enhancement	Stability
Masuda *et al.*^23^	10–20 nm	Al_2_O_3_, SiO_2_	DI water	∼11% varies with concentration	Moderate to low without surfactant
Duangthongsuk & Wongwises^34^	21 nm avg.		DI water	Increased with temperature and concentration	Stable during experimental period
Turgut *et al.*^35,38^	10–25 nm		DI water	Increased with concentration; minimal temp. effect	Stable during test period
Reddy & Rao^36^	20–30 nm		DI water	∼0.6–14.2% depending on wt% and fluid	Good even without surfactant
			Water/EG		
Saleh *et al.*^37^	40 nm		DI water	Significant with SDS surfactant	Improved with surfactant
Khedkar *et al.*^21^	25–50 nm		EG	∼15–20%	Stable over 3 weeks
Moradi *et al.*^30^	Not specified	MWCNTs	DI water	36.3% at 50 °C, 1.12 vol% concentration	2 weeks
			Water/EG		
Esfe *et al.*^43^	Not specified	MWCNTs	DI water	34.31% at 60 °C, 1 vol% concentration	Stable under experimental condition
			Water/EG		
Akhgar *et al.*^44,45^	10–25 nm	COOH-functionalized MWCNTs	DI water	38.7% at 50 °C 0.05–1 vol% concentrations	Not mentioned, used CTAB surfactant
			Water/EG		
Current study	15 and 30 nm	COOH-functionalized MWCNTs	EG	∼8.8–16.7% (size and concentration dependent)	More than 4 weeks for small size

For example, Khedkar *et al.*^21^ achieved 15–20% enhancement with 15–35 nm TiO_2_ in deionized (DI) water, while Masuda *et al.*^23^ observed 10–30% improvement with size-dependent behavior. Duangthongsuk and Wongwises^34^ reported stable 21 nm TiO_2_ dispersions in DI water, and Turgut *et al.*^35^ found a 7.4% increase using 10–25 nm particles. Reddy and Rao^36^ noted 0.6–14.2% enhancement for 20–30 nm TiO_2_ in water/EG mixtures without surfactants, while Saleh *et al.*^37^ observed additional improvement with SDS. Maheshwari *et al.*^38^ investigated how TiO_2_–water nanofluids were affected by concentration, particle size, and shape. Although the durability of these nanofluids was not examined, they discovered that high concentrations (∼2.5 wt%) of the cubic-shaped nanoparticles provide a high thermal conductivity. In their investigation of TiO_2_ nanofluids in DI water, Azari *et al.*^39^ reported 8.2% improvement in TiO_2_–water systems. Das *et al.*^40^ investigated TiO_2_ (anatase) nanofluids and demonstrated that the surfactants cetyltrimethylammonium bromide (CTAB) and sodium dodecyl sulfate (SDS) provided markedly better colloidal stability compared to acetic acid and sodium dodecyl benzene sulfonate (SDBS). The highest thermal-conductivity enhancement of 5.8% was observed for the SDS-stabilized nanofluid at a 1 vol% loading. Sonawane *et al.*^41^ dispersed TiO_2_ (anatase) nanoparticles in various base fluids, including water, ethylene glycol (EG), and paraffin oil, and reported that TiO_2_/water nanofluids exhibited a 22% higher thermal conductivity than those based on other fluids. Azmi *et al.*^42^ further examined TiO_2_ nanofluids in a water–EG mixture and achieved a maximum enhancement of 15.4% at 1.5 vol%.

Despite these advances, issues such as long-term stability, environmental and health risks of nanoparticles, and the high cost of large-scale production remain challenges. To overcome these limitations, recent studies have explored hybrid nanofluids, which combine different nanoparticles or base fluids to improve both stability and heat-transfer performance. Among these, hybrids composed of MWCNTs and TiO_2_ have attracted particular interest due to their synergistic behavior. For instance, Esfe *et al.*^43^ investigated MWCNT–TiO_2_ (70 : 30) nanofluids in an EG–water mixture and reported a 36.3% thermal conductivity enhancement at 50 °C and 1.12 vol%. Akhgar *et al.*^44,45^ investigated the thermal conductivity of hybrid TiO_2_/MWCNT nanofluids dispersed in a water–ethylene glycol mixture and reported enhancements of up to 38.7% at nanoparticle volume fractions between 0.05% and 1%. Similarly, Moradi *et al.*^30^ examined TiO_2_/MWCNT/EG–water hybrid nanofluids and found a maximum conductivity gain of 34.3% at 60 °C and 1 vol% concentration.

In spite of extensive research, challenges remain, particularly in maintaining nanofluid stability over time and minimizing particle aggregation at intermediate concentrations. Hybrid nanofluids, composed of multiple types of nanostructures, offer a synergistic strategy to enhance thermal performance while preserving dispersion stability. Among various formulations, composites combining TiO_2_ nanoparticles with multi-walled carbon nanotubes (MWCNTs) have shown exceptional potential. Recent studies^30,43^ have demonstrated that TiO_2_–MWCNT hybrids can achieve remarkable improvements in thermal conductivity due to their complementary heat-transfer mechanisms and strong interfacial interactions.

**Fig. 2:**
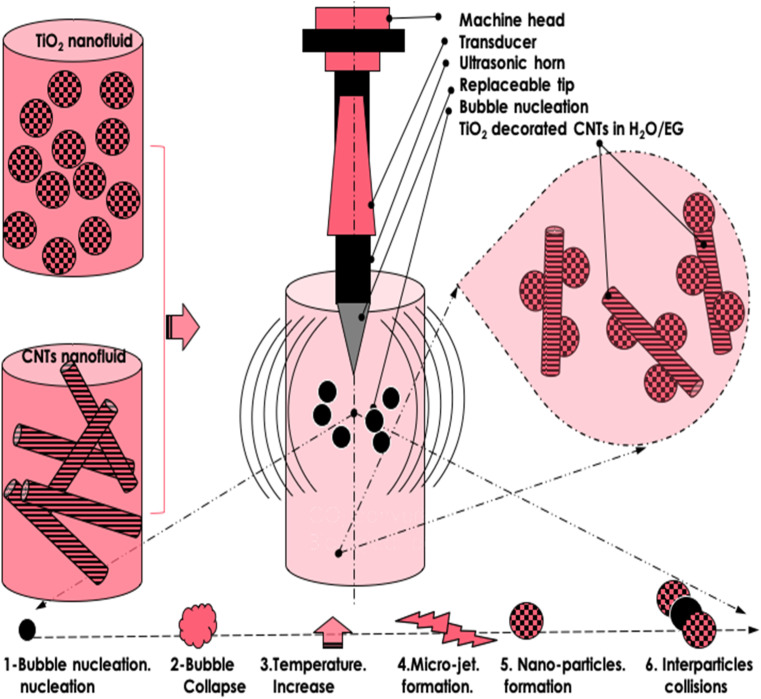
Schematic representation of the 1-step ultrasonication configuration used for the preparation of the TiO_2_-(CH_2_OH)_2_, MWCNT-(CH_2_OH)_2_, and TiO_2_/MWCNT-(CH_2_OH)_2_ nanofluids.

As discussed earlier, the TiO_2_/MWCNT-ethylene glycol nanofluids with smaller TiO_2_ nanoparticles exhibited an average thermal-conductivity enhancement of approximately 16.7%, together with temporal stability exceeding four weeks. The principal advantage of the ultrasonication method lies in its scalability for mass production while minimizing nanoparticle clustering or aggregation, even without surfactants. As schematically illustrated in Fig. 2, this desirable behavior originates from the intrinsic cavitation dynamics occurring during probe ultrasonication. The process begins with bubble nucleation and rapid collapse, generating localized high-energy zones and transient temperature spikes. Subsequent microjet formation and shockwave propagation promote nanoparticle fragmentation, uniform dispersion, and strong interfacial mixing within the base fluid. The energetic interparticle collisions produced during this process further inhibit agglomeration, resulting in a homogeneous and stable hybrid nanofluid suitable for industrial-scale thermal applications.

Despite substantial progress on TiO_2_ and hybrid nanofluids, several critical gaps remain: (i) achieving significant (>10%) thermal-conductivity enhancement at ultra-low solid contents (<0.02 wt%), (ii) maintaining multi-week stability in ethylene glycol without surfactants, and (iii) quantifying the influence of particle size at fixed MWCNT loading under an industrially scalable probe-ultrasonication process. This study aim to address these gaps through a systematic investigation of 15 nm *versus* 30 nm TiO_2_ in hybrid TiO_2_/MWCNT nanofluids prepared *via* probe ultrasonication.

In contrast to previous studies, the present work introduces a novel formulation strategy employing highly dilute hybrid nanofluids, consisting of a fixed ultra-low concentration of MWCNTs (0.001 wt%) combined with TiO_2_ at 0.001 and 0.01 wt%. These compositions correspond to overall volume fractions of approximately 0.0008% and 0.0033%, respectively. Remarkably, despite such extremely low nanoparticle loadings, thermal conductivity enhancements of up to 16.7% at 70 °C were achieved, accompanied by stability exceeding four weeks. To the best of our knowledge, this represents the first report demonstrating such a substantial improvement in an ethylene glycol-based TiO_2_/MWCNT hybrid nanofluid at sub-0.01 wt% concentrations, achieved through a surfactant-free and scalable ultrasonication route while maintaining excellent long-term colloidal stability.

## 2. Experiments and results

By decorating multi-walled carbon nanotubes (MWCNTs) with titanium dioxide (TiO_2_) nanoparticles of two distinct diameters (15 nm and 30 nm) in an ethylene glycol (CH_2_OH)_2_ base fluid, TiO_2_-decorated MWCNT hybrid nanofluids were synthesized to investigate the influence of TiO_2_ particle size on thermal conductivity enhancement and temporal stability. The optimized formulation of 15 nm TiO_2_-decorated MWCNTs in ethylene glycol, exhibited a reproducible thermal conductivity improvement of 16.7% and maintained colloidal stability for 30 days.

### 2.1. Materials & methods

The morphology, structural integrity, and dispersion quality of the synthesized nanofluids were confirmed through comprehensive analyses employing Raman spectroscopy, transmission electron microscopy (TEM), and UV-Vis spectrophotometry. Thermal conductivity was measured using the transient hot-wire (THW) method across a temperature range of 20–70 °C, revealing significant improvements compared with the base fluid. A maximum enhancement of 16.7% was observed for the hybrid nanofluid containing 15 nm TiO_2_ nanoparticles at 70 °C. Furthermore, UV-Vis spectroscopy and zeta potential measurements demonstrated excellent long-term dispersion stability, with the hybrid nanofluids maintaining uniform suspension for more than one month—particularly at smaller particle sizes and lower concentrations.

Carboxylic acid-functionalized multi-walled carbon nanotubes (MWCNT-COOH) were procured from US Research Nanomaterials, Inc. They were synthesized *via* the chemical vapor deposition (CVD) method, and the degree of surface functionalization (–COOH ≈ 2.3 at%) was verified by FTIR spectroscopy and the supplier's certificate of analysis. Titanium dioxide (TiO_2_, anatase phase, 15 nm and 30 nm) was also obtained from the same supplier. The specific surface area (SSA) was determined using the Brunauer–Emmett–Teller (BET) method. All materials were used as received without further purification or surface treatment. Their physical and morphological characteristics are summarized in Table 2.

**Table 2:** The characteristics of nanomaterials

Nano material	Density (g cm^−3^)	Purity (%)	SSA (m^2^ g^−1^)	Diameter (nm)	Length (µm)	Color	Morphology
MWCNT	2.1	>95%	60	External: 30–50 internal: 5–12	10–30	Black	Cylindrical with standard shape anisotropy
TiO_2_, anatase	3.9	>99%	60	15	—	White	Quasi spherical
TiO_2_, anatase	3.9	>99%	50	30	—	White	Quasi spherical

The purpose of employing these nanomaterials was to develop hybrid nanofluids on a scalable basis for potential industrial heat-transfer applications. The strong interfacial bonding and excellent dispersion behavior of functionalized multi-walled carbon nanotubes (MWCNTs) in polar base fluids make them suitable scaffolds for forming stable nanocomposites. Furthermore, the quasi-spherical morphology of TiO_2_ nanoparticles can contribute to a reduced pressure drop in heat-exchange systems, whereas the one-dimensional structure of MWCNTs facilitates phonon transport and thereby enhances the effective thermal conductivity.^46^

Prior to the main probe-ultrasonication step (Fig. 2), both MWCNTs and TiO_2_ nanoparticles were first dispersed separately in ethylene glycol using a mechanical stirrer under controlled temperature conditions to achieve uniform pre-mixing. A thermostatically regulated water bath with digital temperature control was used to maintain a constant temperature during the process. The two suspensions were then combined and stirred for 10 min to ensure homogeneous blending.

Subsequently, the hybrid suspension was subjected to probe ultrasonication to disrupt any residual agglomerates and promote TiO_2_ decoration onto the MWCNT surfaces.

The hybrid nanofluid was sonicated using a Sonics & Materials VCX-750 ultrasonic processor (750 W, 20 kHz) equipped with a 13 mm titanium probe, operated at 20% amplitude in pulsed mode (2 s on/2 s off) for 5 min. The dispersion temperature was maintained below 30 °C using a thermostatic water bath. These parameters ensured uniform cavitation energy distribution and reproducibility of nanoparticle dispersion.

During probe ultrasonication, acoustic cavitation generates localized microjets and shear forces that break up nanoparticle clusters, producing homogeneous and stable nanofluids. This mechanism simultaneously facilitates the surface decoration of MWCNTs with TiO_2_ nanoparticles, as confirmed by Raman spectroscopy.

**Fig. 3:**
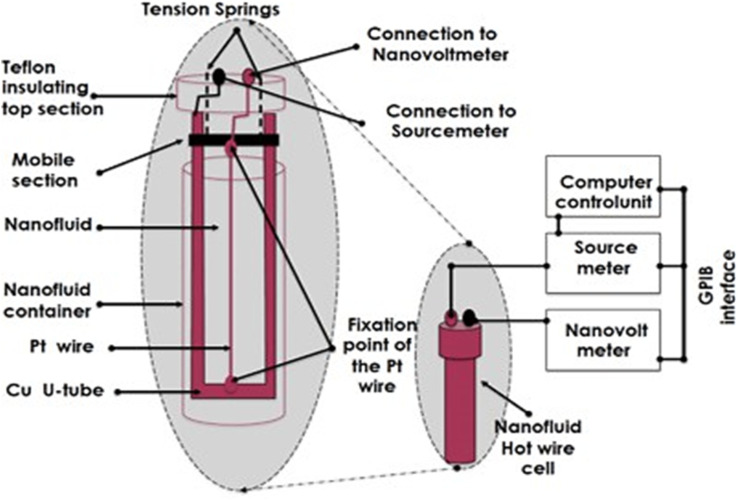
Schematic representation of the transient hot-wire setup used for thermal conductivity measurements.

In the present work, dilute hybrid nanofluids were prepared with a fixed MWCNT concentration of 0.001 wt% and TiO_2_ concentrations of 0.001 wt% and 0.01 wt%. Two TiO_2_ particle sizes (15 nm and 30 nm) were examined. Characterization included transmission electron microscopy (TEM), Raman spectroscopy, dynamic light scattering (DLS), and UV-Vis spectroscopy. Thermal-conductivity measurements were performed using the standard transient hot-wire method, schematically illustrated in Fig. 3.

### 2.2. Morphological investigations

The size, shape, and spatial distribution of nanoparticles strongly influence the thermal behavior of nanofluids. To assess these features, transmission electron microscopy (TEM) was employed to examine the morphology and dispersion of the synthesized TiO_2_/MWCNT-(CH_2_OH)_2_ hybrid nanocomposites.

**Fig. 4:**
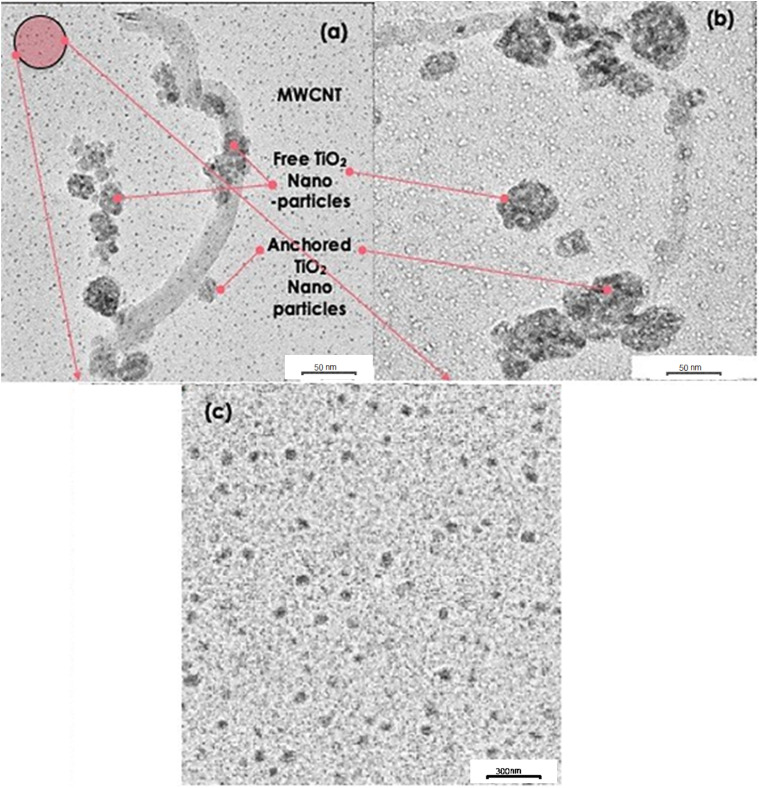
Transmission electron microscopy (TEM) images of TiO_2_-decorated MWCNT/EG hybrid nanofluids: (a) 15 nm TiO_2_, (b) 30 nm TiO_2_, and (c) magnified region of 15 nm TiO_2_.

Fig. 4 presents TEM images of two representative samples containing TiO_2_ nanoparticles of 15 nm (a) and 30 nm (b) average diameters. In both cases, three distinct populations of TiO_2_ nanoparticles can be identified: (i) TiO_2_ nanoparticles uniformly anchored onto MWCNT surfaces, (ii) unanchored TiO_2_ agglomerates, and (iii) well-dispersed, isolated TiO_2_ nanoparticles. Notably, the population density of well-dispersed nanoparticles is markedly higher in the 15 nm TiO_2_/MWCNT-(CH_2_OH)_2_ hybrid compared to the 30 nm counterpart, indicating improved surface anchoring and dispersion homogeneity at smaller particle sizes.

### 2.3. Vibrational Raman investigations

**Fig. 5:**
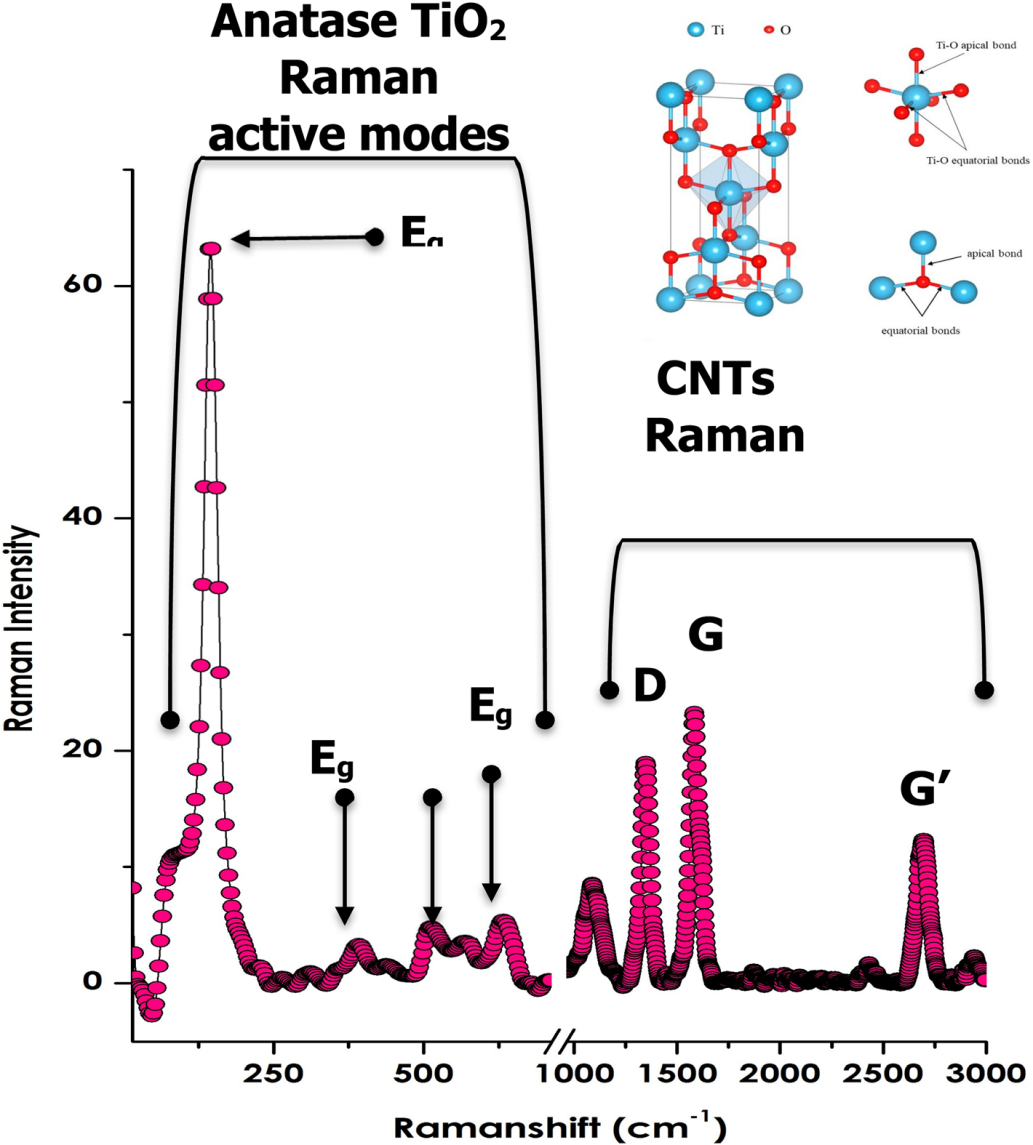
Raman spectrum of TiO_2_/MWCNT (15 nm) nanocomposite at room temperature.

Fig. 5 shows the room-temperature Raman spectrum of the TiO_2_-decorated MWCNT/EG hybrid nanofluid (15 nm TiO_2_). The spectrum exhibits two main groups of Raman-active modes. The first group, with peaks at 142.5, 394.6, 508.8, and 567.3 cm^−1^, corresponds to the characteristic vibrational modes of anatase-phase TiO_2_. The second group, with peaks at 1345.3, 1586.9, and 2693.5 cm^−1^, arises from the D, G, and 2D bands of graphitic carbon, confirming the structural integrity of the MWCNT framework.

An additional peak near 1083.8 cm^−1^ is also observed, which can be attributed to surface vibrational (Ti–O–C) modes formed between the –COOH functional groups of MWCNTs and surface hydroxyl groups of TiO_2_.^47,48^ The presence of this interfacial Ti–O–C linkage indicates strong chemical coupling between TiO_2_ nanoparticles and MWCNTs, enhancing interfacial adhesion and minimizing particle detachment. This robust bonding contributes significantly to the long-term colloidal stability of the hybrid nanofluid, consistent with the high zeta potential values (>30 mV) obtained in this study.

### 2.4. UV-Vis absorbance and time stability investigations

**Fig. 6:**
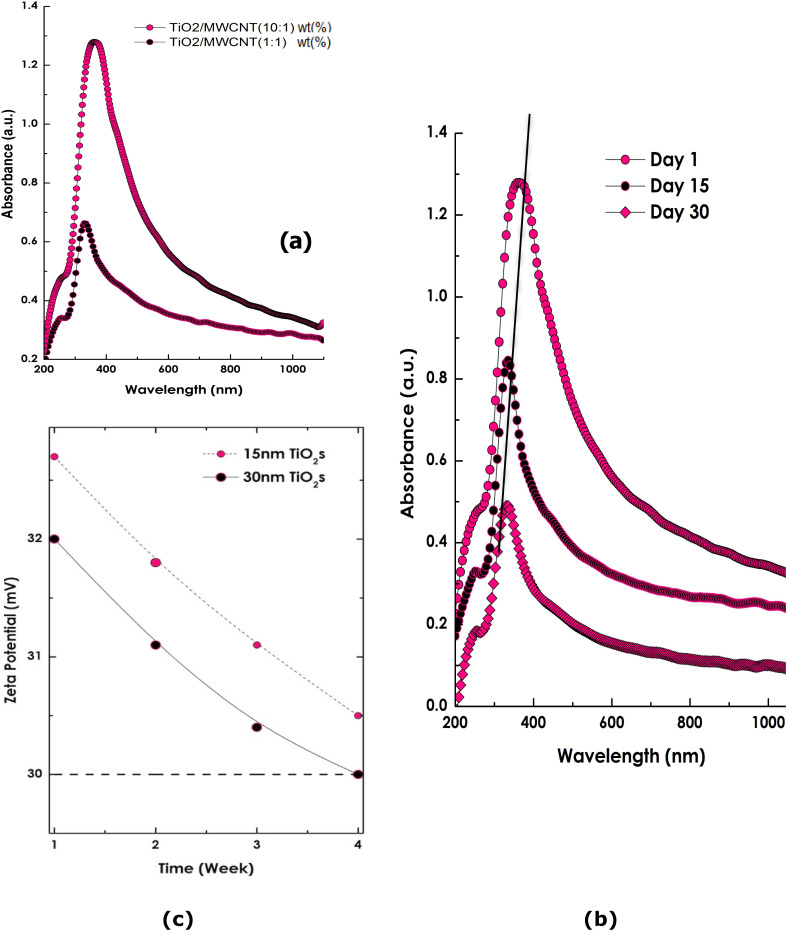
(a) Optical absorbance spectra of TiO_2_/MWCNT-EG nanofluids at different TiO_2_ loadings (MWCNT : TiO_2_ = 10 : 1 and 1 : 1 wt%) using 15 nm TiO_2_. (b) Time-evolution of the optical spectra for TiO_2_ (15 nm)/MWCNT-EG (1 : 1 wt%) measured on days 1, 15, and 30. (c) Variation of zeta potential over time for 15 nm and 30 nm TiO_2_/MWCNT-EG hybrid nanofluids.

To optimize the TiO_2_ loading on MWCNTs and determine the influence of their relative concentrations in ethylene glycol (EG), hybrid nanofluids with different TiO_2_ contents were prepared. Fig. 6a presents the UV-Vis absorbance spectra of TiO_2_/MWCNT hybrid nanofluids synthesized with a fixed MWCNT concentration of 0.001 wt% and TiO_2_ concentrations of 0.001 wt% and 0.01 wt%, using TiO_2_ particles of 15 and 30 nm in size.

The spectra are dominated by the characteristic absorption of TiO_2_ in the 300–400 nm range, corresponding to intrinsic electronic transitions of the TiO_2_ nanoparticles. A secondary feature is observed in the 200–300 nm region (UV-blue shoulder), which is attributed to the π–π* and/or σ–σ* electronic transitions of the MWCNTs. The presence of both signatures confirms the successful hybridization of TiO_2_ with the MWCNT matrix.

To assess the colloidal stability, UV-Vis spectra of the TiO_2_ (15 nm)/MWCNT-EG nanofluid were recorded at different storage intervals (1, 15, and 30 days), as shown in Fig. 6(b). The overall spectral profiles remain consistent, exhibiting the characteristic π–π* and σ–σ* transitions of MWCNTs alongside the dominant TiO_2_ absorption peak. The TiO_2_ absorption maxima were observed to shift from 361.5 to 337.6 and finally to 330.9 nm over the 30-day period, accompanied by a moderate decrease in intensity. This gradual blue-shift, together with the preserved spectral profile, indicates minimal sedimentation and sustained nanoparticle dispersion.

These observations are consistent with the zeta potential results shown in Fig. 6(c), which reveal surface-charge values exceeding +30 mV for the TiO_2_/MWCNT (15 nm)-EG hybrid nanofluid, confirming excellent electrostatic stabilization. Overall, the hybrid nanofluid demonstrates appreciable time stability for at least four weeks under ambient conditions, in agreement with the observed spectral and electrokinetic behavior.

### 2.5. Thermal conductivity enhancement investigations

The thermal conductivity of the hybrid nanofluids was measured in the temperature range of 20–70 °C using the transient hot-wire (THW) method, as schematically illustrated in Fig. 3. In this technique, a thin metallic wire immersed in the nanofluid is heated by a constant current pulse, and the transient temperature rise of the wire is recorded. From the slope of the temperature–time response, the thermal conductivity of the surrounding medium can be determined with high precision.

The relative enhancement of the hybrid nanofluid with respect to the base fluid was calculated using the following relation:1
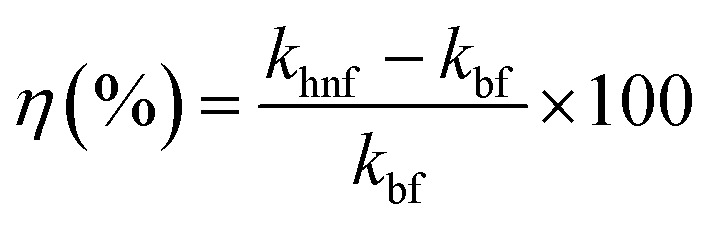
where, *k*_bf_ and *k*_hnf_ are the thermal conductivities of the base fluid (ethylene glycol) and the hybrid nanofluid, respectively.

The viscosity of nanofluids generally decreases with increasing temperature, leading to higher molecular mobility and kinetic energy. Consequently, the frequency of collisions between nanoparticles and fluid molecules increases, enhancing heat transport *via* intensified Brownian motion and micro-convection mechanisms.

**Fig. 7:**
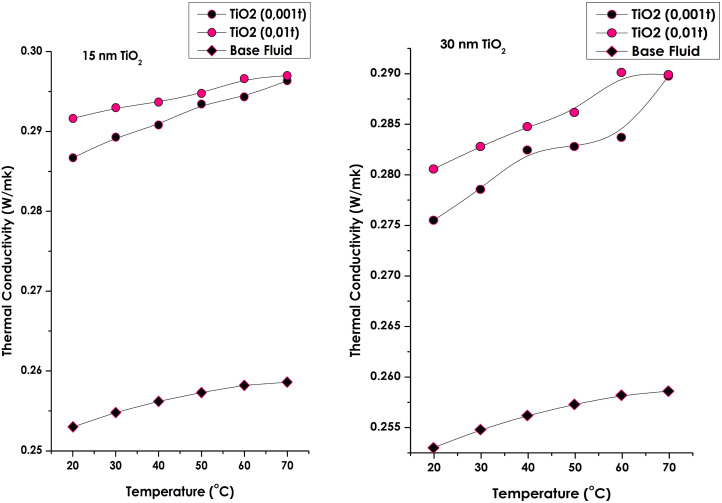
Thermal conductivity of the optimized TiO_2_ (15 nm, 30 nm/0.01 and 0.001 wt%)/MWCNTs (0.001 wt%) nano fluids.

Fig. 7 illustrates the temperature dependence of the thermal conductivity for hybrid nanofluids containing TiO_2_ nanoparticles of two sizes (15 nm and 30 nm) and at two concentrations (0.001 wt% and 0.01 wt%), while maintaining a constant MWCNT loading of 0.001 wt%. A clear and systematic increase in thermal conductivity is observed for all samples with rising temperature. The enhancement is most pronounced at higher TiO_2_ concentration (0.01 wt%), particularly for the smaller 15 nm particles, which exhibit the steepest conductivity gain with temperature.

**Fig. 8:**
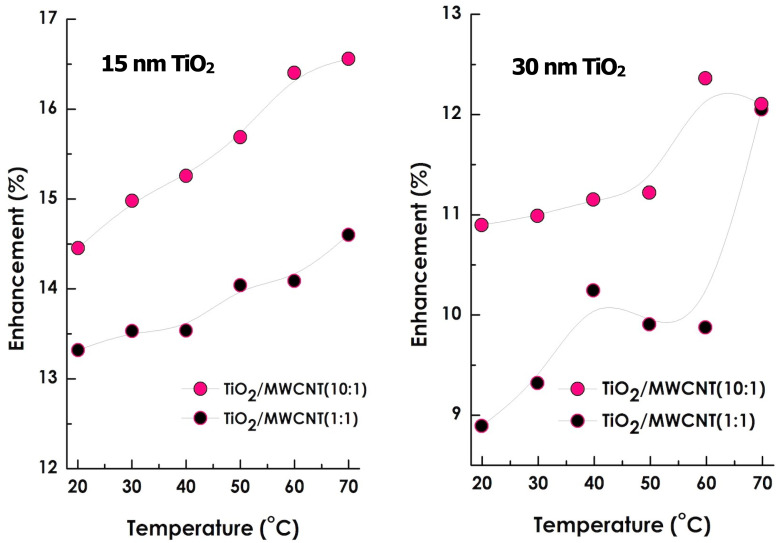
Thermal conductivity enhancement of the optimized TiO_2_ (15 nm, 30 nm/0.01 & 0.001 wt%)/MWCNTs (0.001wt%) nano fluids.

As summarized in Table 3 and shown in Fig. 8, all TiO_2_/MWCNT hybrid nanofluids demonstrated a monotonic rise in thermal conductivity with both increasing temperature and TiO_2_ concentration. The hybrid nanofluid containing 15 nm TiO_2_ at 0.01 wt% achieved the maximum enhancement of approximately 16.7% at 70 °C. This improvement is primarily attributed to (i) the larger specific surface area of smaller TiO_2_ nanoparticles, which promotes stronger phonon coupling across the TiO_2_/MWCNT interfaces, and (ii) enhanced Brownian motion at elevated temperatures.

**Table 3:** Thermal conductivity enhancement parameters of the various samples

Hybrid decorated MWCNT nanofluid with various TiO_2_	Concent. (wt%)	Size (nm)	Thermal conductivity (25 °C) W m^−1^ K^−1^	Thermal conductivity (70 °C) W m^−1^ K^−1^	Thermal conductivity enhancement (25 °C) (%)	Thermal conductivity enhancement (70 °C) (%)	Average thermal conductivity enhancement (%)
TiO_2_, anatase	0.001	30	0.2770	0.2897	9.11	12.04	10.58
TiO_2_, anatase	0.01	30	0.2816	0.2899	10.94	12.10	11.52
TiO_2_, anatase	0.001	15	0.2879	0.2963	14.50	16.70	15.60
TiO_2_, anatase	0.01	15	0.2923	0.2969	15.12	14.85	14.98

In contrast, samples containing 30 nm TiO_2_ exhibited lower enhancements, likely due to their reduced effective interfacial contact area and weaker phonon transport efficiency. These results confirm that smaller nanoparticles create more efficient thermal conduction pathways while maintaining excellent colloidal stability over a four-week period in the absence of surfactants. Overall, the findings highlight the synergistic role of nanoscale interfacial coupling and Brownian micro-convection in achieving superior heat-transfer performance in hybrid TiO_2_/MWCNT-EG nanofluids.

At a constant temperature, the thermal conductivity of the nanofluid increases with rising TiO_2_ concentration and decreasing TiO_2_ particle size. Higher particle concentrations promote the formation of interconnected nanoparticle clusters, which facilitate heat transfer through solid–solid contact pathways rather than through the less conductive liquid medium. Consequently, a significant enhancement in thermal conductivity can be achieved by simultaneously increasing TiO_2_ concentration and temperature while reducing nanoparticle size. Each measurement was repeated twice to ensure reproducibility, and the mean values were reported. The thermal conductivity of pure ethylene glycol was also measured to validate the experimental setup, showing excellent agreement with reference data reported in the literature.^49^

The superior conductivity observed for the smaller (15 nm) TiO_2_ nanoparticles originates from their higher specific surface area and the lower interfacial (Kapitza) thermal resistance between TiO_2_ and MWCNT surfaces. The increased surface-to-volume ratio enhances phonon coupling and interfacial heat exchange. Additionally, smaller nanoparticles exhibit stronger Brownian motion, generating localized micro-convection that further promotes energy transport within the ethylene glycol matrix.^16,17,22^

These findings are consistent with recent studies emphasizing the pivotal role of MWCNTs as conductive bridges in hybrid nanofluids. He *et al.*^50^ and Mai *et al.*^51^ demonstrated that the formation of CNT-based percolation networks and oxide-carbon interfaces effectively reduce Kapitza resistance and enhance phonon transport efficiency. Their observations corroborate the present results, confirming that TiO_2_/MWCNT interfacial coupling and the creation of hybrid phonon pathways significantly improve heat-transfer performance, even at ultra-low nanoparticle loadings.

## 3. Conclusion

This study demonstrates a substantial improvement in both thermal conductivity and dispersion stability of TiO_2_/MWCNT hybrid nanofluids synthesized *via* a scalable probe-ultrasonication method. The formulation containing 15 nm TiO_2_ at 0.01 wt% achieved the highest thermal-conductivity enhancement of approximately 16.7% at 70 °C, while maintaining excellent colloidal stability for over four weeks.

The superior performance of smaller TiO_2_ nanoparticles arises from their larger specific surface area, stronger Ti–O–C interfacial bonding with carboxyl-functionalized MWCNTs, and lower interfacial (Kapitza) thermal resistance. Probe ultrasonication proved to be a cost-effective, surfactant-free, and industrially scalable technique for producing homogeneous and stable hybrid nanofluids. Complementary zeta-potential and UV-Vis spectroscopy analyses further confirmed the remarkable long-term stability, particularly at lower nanoparticle sizes and concentrations.

This study demonstrates a scalable, surfactant-free route achieving 16.7% conductivity enhancement at 0.01 wt% TiO_2_ (15 nm). The method's industrial potential lies in its reproducibility and low-solid content. Future studies should address viscosity and long-term stability beyond 70 °C for deployment in high-temperature heat-transfer systems.

## Conflicts of interest

The authors declare no conflict of interest.

## Data Availability

All data generated during this study is included in the manuscript.
